# Proteoglycan-4 regulates fibroblast to myofibroblast transition and expression of fibrotic genes in the synovium

**DOI:** 10.1186/s13075-020-02207-x

**Published:** 2020-05-13

**Authors:** Marwa Qadri, Gregory D. Jay, Ling X. Zhang, Holly Richendrfer, Tannin A. Schmidt, Khaled A. Elsaid

**Affiliations:** 1 Department of Biomedical and Pharmaceutical Sciences, Chapman University School of Pharmacy, Rinker Health Sciences Campus, 9401 Jeronimo Road, Irvine, CA 92618 USA; 2 Department of Pharmacology, College of Pharmacy, Jazan University, Jazan, 82826 Saudi Arabia; 3 Department of Emergency Medicine, Rhode Island Hospital, Providence, RI USA; 4 Biomedical Engineering Department, School of Dental Medicine, University of Connecticut, Farmington, CT USA

**Keywords:** Proteoglycan-4, CD44, SMA, Synovial fibrosis, Myofibroblast

## Abstract

**Background:**

Synovial tissue fibrosis is common in advanced OA with features including the presence of stress fiber-positive myofibroblasts and deposition of cross-linked collagen type-I. Proteoglycan-4 (PRG4) is a mucinous glycoprotein secreted by synovial fibroblasts and is a major component of synovial fluid. PRG4 is a ligand of the CD44 receptor. Our objective was to examine the role of PRG4-CD44 interaction in regulating synovial tissue fibrosis in vitro and in vivo.

**Methods:**

OA synoviocytes were treated with TGF-β ± PRG4 for 24 h and α-SMA content was determined using immunofluorescence. Rhodamine-labeled rhPRG4 was incubated with OA synoviocytes ± anti-CD44 or isotype control antibodies and cellular uptake of rhPRG4 was determined following a 30-min incubation and α-SMA expression following a 24-h incubation. HEK-TGF-β cells were treated with TGF-β ± rhPRG4 and Smad3 phosphorylation was determined using immunofluorescence and TGF-β/Smad pathway activation was determined colorimetrically. We probed for stress fibers and focal adhesions (FAs) in TGF-β-treated murine fibroblasts and fibroblast migration was quantified ± rhPRG4. Synovial expression of fibrotic markers: α-SMA, collagen type-I, and PLOD2 in *Prg4* gene-trap (*Prg4*^*GT*^) and recombined *Prg4*^*GTR*^ animals were studied at 2 and 9 months of age. Synovial expression of α-SMA and PLOD2 was determined in 2-month-old *Prg4*^*GT/GT*^&*Cd44*^*−/−*^ and *Prg4*^*GTR/GTR*^&*Cd44*^*−/−*^ animals.

**Results:**

PRG4 reduced α-SMA content in OA synoviocytes (*p < 0*.*001*). rhPRG4 was internalized by OA synoviocytes via CD44 and CD44 neutralization attenuated rhPRG4’s antifibrotic effect (*p < 0*.*05*). rhPRG4 reduced pSmad3 signal in HEK-TGF-β cells (*p < 0*.*001*) and TGF-β/Smad pathway activation (*p < 0*.*001*). rhPRG4 reduced the number of stress fiber-positive myofibroblasts, FAs mean size, and cell migration in TGF-β-treated NIH3T3 fibroblasts (*p < 0*.*05*). rhPRG4 inhibited fibroblast migration in a macrophage and fibroblast co-culture model without altering active or total TGF-β levels. Synovial tissues of 9-month-old *Prg4*^*GT/GT*^ animals had higher α-SMA, collagen type-I, and PLOD2 (*p < 0*.*001*) content and *Prg4* re-expression reduced these markers (*p < 0*.*01*). *Prg4* re-expression also reduced α-SMA and PLOD2 staining in CD44-deficient mice.

**Conclusion:**

PRG4 is an endogenous antifibrotic modulator in the joint and its effect on myofibroblast formation is partially mediated by CD44, but CD44 is not required to demonstrate an antifibrotic effect in vivo.

## Background

Osteoarthritis (OA) is the most prevalent type of musculoskeletal disorder in patients 60 years or older [1–3]. OA is a leading cause of pain and disability and patients with symptomatic knee OA are at an increased risk of all-cause mortality [4–6]. The etiology of OA is complex where joint trauma, obesity, and genetics, among other risk factors, contribute to disease development [7–10]. OA affects the whole joint with manifestations that include degeneration of articular cartilage and the meniscus, abnormal bone remodeling, and synovial inflammation [11–13]. Macroscopic evidence of synovial inflammation or synovitis is a common finding in up to 74% of patients with knee OA of different grades and 95% of patients with moderate to severe OA [14–16]. OA synovitis is likely caused by an innate immune response, mediated by the toll-like receptors 2 and 4 (TLR2 and TLR4) in the synovium, and resultant expression of inflammatory cytokines, chemokines, and matrix-degrading enzymes [17–21]. The extent of synovitis is a strong predictor of OA progression across multiple studies, and therefore treating synovial inflammation is a potentially important target for therapeutic intervention, especially during the early stage of OA [14, 22–27].

The normal synovium is composed of a cellular intimal layer and a subintimal connective tissue layer [14, 28]. The intimal layer is usually 1–4 cells in thickness with two types of cells: type A macrophages and type B synovial fibroblasts, with macrophages making up a smaller percentage of cells in the intimal layer of the healthy synovium [28]. Synovial fibroblasts synthesize hyaluronic acid (HA), a glycosaminoglycan, and proteoglycan-4 (PRG4), a mucinous glycoprotein, which are major components of synovial fluid (SF) with important roles in joint lubrication [29, 30]. In synovitis, synovial membranes become hyperplastic with increased accumulation of macrophages and other inflammatory cells, along with subintimal fibrosis and angiogenesis [13, 14, 31]. Synovial fibrosis can be considered a maladaptive healing response to chronic synovitis which is characterized by the upregulation of collagen type-I expression, fibroblast transition to a myofibroblast-like phenotype through de novo expression of alpha smooth muscle actin (α-SMA) and enhanced migratory behavior, and upregulation of procollagen-lysine, 2-oxoglutarate 5-dioxygenase 2 (PLOD2) leading to accumulation of cross-linked collagen in the synovium [32–34]. These pathological changes are induced in the OA synovium by transforming growth factor beta (TGF-β1) and its associated signaling pathways [35, 36]. Synovial fibrosis also contributes to joint pain and stiffness observed in advanced OA [31, 37].

In a recent study, we demonstrated that increasing intracellular adenosine 3′, 5′-cyclic monophosphate (cAMP), using forskolin, reduced collagen type-I, PLOD2, and α-SMA expression in TGF-β-stimulated osteoarthritic fibroblast-like synoviocytes (OA FLS) [38]. Associated with this antifibrotic effect was an increase in HA and PRG4 production by OA FLS, whereby native synovial human PRG4 reduced procollagen type-I level in OA FLS [38]. Furthermore, synoviocytes from *Prg4* null mice displayed more extensive collagen type-I staining compared to synoviocytes from *Prg4* competent mice [38]. In a separate study, we have also shown that PRG4 is a ligand for the HA receptor, CD44 [39]. We have also reported that PRG4-CD44 interaction inhibited interleukin-1 beta (IL-1β) induced OA FLS proliferation and expression of matrix-degrading enzymes [40], via the inhibition of nuclear factor kappa b (NF-κB) nuclear translocation mediated by blocking inhibitory kappa b (IκB) degradation [40].

It remains unknown whether PRG4 has a role in regulating fibroblast to myofibroblast transition and associated cell migration in the fibrotic synovium. Furthermore, it is yet to be determined whether PRG4 regulates synovial fibrosis in vivo and whether this role is due to its interaction with the CD44 receptor. Using recombinant human proteoglycan-4 (rhPRG4), we aimed to study the role of PRG4 in regulating fibroblast to myofibroblast transition and modulating fibroblast migration in response to exogenous TGF-β or co-incubation with lipopolysaccharide (LPS) stimulated macrophages. We also studied the role of PRG4-CD44 interaction, and more specifically CD44-mediated cellular uptake of PRG4, in the regulation of myofibroblast formation in vitro and progression of synovial fibrosis in vivo. We hypothesized that PRG4 regulates fibroblast to myofibroblast transition and prevents synovial fibrosis in a CD44-dependent manner.

## Methods

### Impact of PRG4 and HA treatments on *ACTA2* expression, α-SMA immunostaining, and stress fiber formation in osteoarthritic fibroblast-like synoviocytes (OA FLS) and the role of CD44 in mediating the effect of rhPRG4 in TGF-β-stimulated OA FLS

OA FLS (Cell Applications, USA) were isolated from synovial tissues from de-identified patients undergoing knee replacement surgery (*n* = 4; 2 males and 2 females; 62–69 years old). Cells were received in their second passage and were cultured as previously described [20]. OA FLS were used between the 3rd and 6th passages to avoid alterations in gene expression pattern and cell proliferation rate [41]. OA FLS (300,000 cells/well) in serum-free DMEM media were treated with recombinant human TGF-β1 (R&D Systems, USA) (1 ng/mL) ± native human synovial PRG4 (apparent mol mass 280 kDa as a monomer; 100 μg/mL) [42] or high-molecular weight hyaluronic acid (HA; ~ 1200 kDa; R&D Systems; 100 μg/mL) for 24 h. RNA isolation, cDNA synthesis, and quantitative PCR were performed as previously described [38]. The cycle threshold (Ct) value of α-SMA (*ACTA2*) was normalized to the Ct value of *GAPDH* in the same sample, and the relative expression was calculated using the 2^−ΔΔCt^ method [43]. All primers and probes utilized in our study are commercially available (Thermo Fisher Scientific, USA).

Assessment of α-SMA content in OA FLS was conducted using immunofluorescence and determination of corrected total cell fluorescence (CTCF) using a Nikon E600 fluorescence microscope. OA FLS (200,000 cells/well) were cultured on collagen type-I-coated 22 mm glass coverslips for 48 h in DMEM medium + 10% fetal bovine serum (FBS). Cells were treated with TGF-β1 (1 ng/mL) ± PRG4 or HA (100 μg/mL for both treatments) for 48 h in serum-free DMEM medium. Subsequently, cells were fixed in 10% neutral buffered formalin for 15 min and washed twice with phosphate-buffered saline (PBS). Cells were permeabilized using 0.01% Triton X-100 in PBS and blocked using 2% bovine serum albumin (BSA; Sigma-Aldrich, USA) in PBS for 2 h at room temperature. Probing for α-SMA was performed using FITC-conjugated anti-α-SMA antibody (1:100 dilution; Abcam, USA) and counterstained using Alexa Fluor 594-conjugated anti-α-tubulin antibody (1100 dilution: Abcam) overnight at 4 °C. Following washing with PBS, cells were mounted on DAPI mounting shield (Abcam) and CTCF was quantified using 4 different fields per slide and mean CTCF was calculated. The presence of stress fibers in OA FLS was also evaluated.

Recombinant human PRG4 (rhPRG4; apparent mol mass 240 kDa) is an endotoxin-free full-length product produced by CHO-M cells (Lubris, Framingham, USA) [44]. Rhodamine labeling of rhPRG4 was performed using the Pierce NHS-Rhodamine Antibody Labeling Kit (Thermo Fisher Scientific). OA FLS (200,000 cells/well) were cultured on collagen type-I-coated cover slips and incubated with rhodamine-rhPRG4 (25 μg/mL) ± anti-CD44 or isotype control (IC) antibodies (2 μg/mL for both antibodies; Abcam) for 30 min. Cells were pre-incubated with the antibodies for 1 h prior to rhPRG4 addition. Subsequently, cells were washed twice with PBS and mounted on DAPI mounting media and CTCF was quantified as described above. In another set of experiments, OA FLS (300,000 cells/well) were treated with TGF-β1 (1 ng/mL) ± rhPRG4 (100 μg/mL) ± anti-CD44 or IC antibodies (2 μg/mL for both antibodies) for 24 h followed by determination of *ACTA2* expression as described above.

### CD44-dependent uptake of rhPRG4, Smad3 phosphorylation, and regulation of TGF-β/Smad pathway activation in TGF-β-stimulated HEK Blue-TGF-β cells

HEK Blue-TGF-β is an engineered reporter cell line produced by transfecting human embryonic kidney (HEK) cells with TGF-β receptor 1 (TGF-β R1), Smad3, and Smad4 genes (Invivogen, USA). TGF-β1-treatment results in phosphorylation of Smad3 (pSmad3) and Smad4 (pSmad4), translocation to the nucleus and expression of secreted alkaline phosphatase (SEAP). The activity of SEAP can be detected colorimetrically in media supernatants using a specific substrate (Quanti Blue) at 620–655 nm (Invivogen). HEK-TGF-β cells (500,000) were plated onto sterile chamber slides and incubated with rhodamine-rhPRG4 (25 μg/mL) for 1 h ± anti CD44 or IC antibodies (2 μg/mL for both antibodies). Cells were pre-incubated with the antibodies for 1 h prior to rhPRG4 addition. Subsequently, cells were washed with PBS and intracellular CTCF was determined as described above. In another set of experiments, rhodamine-labeled rhPRG4 (25 μg/mL) was incubated with HEK-TGF-β cells and CD44 was probed using FITC-conjugated anti-CD44 antibody (Abcam) and CD44 (green) and rhPRG4 (red) co-localization was examined using a confocal microscope.

Immunostaining of pSmad3 was performed using an anti-Smad3 antibody that detects phosphorylated serine residues (1:1000 dilution overnight at 4 °C; Abcam). HEK-TGF-β cells were stimulated with TGF-β1 ± rhPRG4 (150 μg/mL) for 1 h. Subsequently, cells were fixed, washed with PBS, and permeabilized as described above. Following probing with anti-pSmad3 antibody, cells were washed with PBS and incubated with goat anti-rabbit IgG (Alexa Fluor 488) (1:1000 dilution for 1 h at room temperature; Abcam). Cells were subsequently washed with PBS and the green immunofluorescence was quantified across all experimental groups. To investigate the impact of rhPRG4 on TGF-β/Smad pathway activation, HEK-TGF-β cells (100,000 cells/well) were cultured in sterile 96-well plates ± TGF-β1 (1 ng/mL) ± rhPRG4 (50, 100, or 150 μg/mL) for 24 h in Quanti-Blue media and the 655 nm absorbance intensity was determined. Data are presented as fold absorbance intensities across experimental groups normalized to untreated controls.

### TGF-β induction of stress fibers, focal adhesions (FAs) and cell migration in murine NIH3T3 fibroblasts, comparison of stress fibers and FAs in *Prg4*^*+/+*^ and *Prg*^*−/−*^ synovial fibroblasts and role of rhPRG4 in regulating fibroblast migration

Murine fibroblasts (NIH3T3; ATCC; 20,000 cells/well) were cultured on collagen type-I-coated 22-mm glass coverslips for 24 h in DMEM supplemented with 10% bovine calf serum (BCS). NIH3T3 cells were starved in DMEM containing 1% BCS for 24 h. Murine fibroblasts were treated with murine TGF-β1 (1 ng/mL) ± rhPRG4 (200 μg/mL) for 24 h in serum-free DMEM. Subsequently, fibroblasts were fixed using neutral buffered formalin, washed with PBS, permeabilized using 0.1% triton X-100, and probed using an anti-α-SMA (marker of stress fibers; 1:1,000) or anti-vinculin (marker of FA complex; 1:1,000) overnight at 4 °C. After washing with PBS, cells were incubated with goat anti-rabbit IgG (Alexa Fluor® 488) at 1:1000 dilution for 1 h at 4 °C. Rhodamine-labeled phalloidin (cytoskeleton label; 1:1000) was added with the secondary antibody. All antibodies were obtained from Abcam. Cells were washed and mounted with DAPI medium for 2 h and viewed under a confocal microscope. A blinded investigator determined the number of stress fiber-positive fibroblasts. At least 100 cells over at least 5 different fields were evaluated, and the percentage of stress fiber-positive fibroblasts was calculated. Separately, mean fibroblast cell spread area was determined using the NIS-Elements imaging software (Nikon). The number and mean size of FAs per cell were determined using ImageJ software as previously described [45]. Fibroblast migration was determined using a scratch assay. A 1000-μL pipette tip was used to perform a uniform scratch in the confluent NIH3T3 fibroblast monolayer. TGF-β (1 ng/mL) stimulation was performed for 48 h ± rhPRG4 (200 μg/mL). Subsequently, cells were washed and stained (Cell Biolabs) and imaged using an all-in-one fluorescence microscope (Keyence). A region of interest was defined and scratch widths were determined across multiple locations. Cell migration was expressed as percent wound closure across different experimental groups as previously described [46].

The phenotype of *Prg4*^*−/−*^ mice is characterized by synovial membrane hyperplasia, chondrocyte apoptosis, and cartilage surface fibrillation [47]. The *Prg4*^*−/−*^ mouse colony is maintained by Dr. Gregory Jay at Rhode Island Hospital (RIH) and is commercially available (stock no. 025737; JAX, USA). The IACUC committee at RIH approved all animal experiments and all experiments were performed according to all applicable guidelines and regulations. Synoviocyte isolation from *Prg4*^*−/−*^ and *Prg4*^*+/+*^ synovial tissues was performed as previously described [38, 39]. *Prg4*^*−/−*^ and *Prg4*^*+/+*^ synoviocytes were plated onto sterile chamber slides (Thermo Fisher Scientific) and allowed to adhere for 48 h. Subsequently, cells were stained for α-SMA and vinculin as described above. The mean size of FAs per cell in *Prg4*^*−/−*^ and *Prg4*^*+/+*^ synoviocytes was determined as described above. *Prg4*^*−/−*^ and *Prg4*^*+/+*^ synoviocytes were seeded in 6-well plates and a scratch was performed in the confluent cell monolayer. Basal synovial fibroblast migration of both genotypes was quantified over 48 h ± rhPRG4 (200 μg/mL), anti-CD44 (2 μg/mL), or IC (2 μg/mL) antibodies and expressed as percent wound closure [46].

### Generation of active TGF-β in lipopolysaccharide-stimulated murine macrophage J774A and NIH3T3 fibroblast co-culture and impact of rhPRG4 treatment on fibroblast migration

Murine J774A macrophages (ATCC) were cultured in DMEM medium + 10% FBS. Macrophages (300,000 cells in DMEM medium + 10% FBS) were stimulated with lipopolysaccharide (LPS; Invivogen) (5 μg/mL) for 24 h. Subsequently, murine macrophages were washed five times with DMEM medium and transferred to the top chamber of a transwell co-culture system (0.4-μm pore size; Sigma-Aldrich). Murine NIH3T3 cells were seeded in the lower chamber of the transwell system and a scratch was performed as described above. NIH3T3 migration was determined following a 48-h incubation of fibroblasts with macrophages in the co-culture system as described above ± rhPRG4 (200 μg/mL). Active and total TGF-β media levels were determined using an ELISA (R&D Systems).

### Age-dependent expression of fibrotic markers in synovial tissues from *Prg4*^*GT/GT*^ animals and the role of PRG4/CD44 interaction in modulating synovial fibrosis in vivo

The *Prg4* gene-trap (*Prg4*^*GT*^) mouse colony is maintained by Dr. Gregory Jay at RIH and is commercially available (stock no. 025740; JAX) [48]. *Prg4*^*GT*^ animal is a genetically engineered PRG4-deficient mouse where the *Prg4* expression can be restored via CRE-mediated recombination [48]. The *Prg4*^*GT/GT*^ mouse recapitulates the hallmark findings in *Prg4*^*−/−*^ mouse, namely synovial tissue hyperplasia and cartilage surface fibrillations and recombination in 3-week-old animals improved but did not completely normalize joint pathological findings [48]. In our studies, recombination (*Prg4*^*GTR/GTR*^) occurred in 3-week-old animals via intraperitoneal injection of tamoxifen (0.1 mg/g in 100 μL corn oil vehicle) daily for 10 days. We compared gene expression and immunostaining of fibrotic markers: α-SMA, COL1A1 (collagen type-I), and PLOD2 in 2-month-old *Prg4*^*GT/GT*^, 2-month-old *Prg4*^*GTR/GTR*^, 9-month-old *Prg4*^*GT/GT*^, and 9-month-old *Prg4*^*GTR/GTR*^ animals. *ACTA2*, *COL1A1*, and *PLOD2* expression levels in murine synovial tissues were performed as previously described [38]. Tissues from each three consecutive mice were pooled and underwent RNA isolation, generating five pooled samples in each experimental group. Separately, animal joints (*n* = 5 in each group) underwent decalcification and paraffin-embedded sectioning as previously described [48]. Immunostaining of synovial tissues was performed using primary antibodies against α-SMA, COL1A1, or PLOD2 (1:1000 dilutions performed overnight at 4 °C) (All antibodies were purchased from Abcam). Subsequently, sections were washed and incubated with goat anti-rabbit IgG (Alexa Fluor 488) (1:1000 dilution) for 1 h and fluorescence intensities (expressed as lumens per square millimeter) were quantified using a fluorescence microscope.

To appreciate the significance of PRG4 and CD44 interaction in the context of fibrotic markers’ expression in the synovium, *Prg4*^*GT/GT*^ animals were crossed with *Cd44*^*−/−*^ mice (stock no. 005085; JAX) [49] to generate *Cd44*^*−/−*^&*Prg4*^*GT/GT*^ animals. Recombination occurred in 3-week-old animals to generate *Cd44*^*−/−*^&*Prg4*^*GTR/GTR*^ animals as described in the last paragraph. Histological analyses of joints harvested from 2-month-old animals (at least 4 animals per group) were performed as described above, using *Cd44*^*+/+*^*&Prg4*^*GT/GT*^ animals from the same litters as controls. We probed synovial tissues for the following fibrotic markers: α-SMA and PLOD2 using specific primary antibodies (1:1000 dilutions) followed by goat anti-rabbit IgG (Alexa Fluor 488) and quantitation of fluorescence intensities (expressed as lumens per square millimeter) as described above.

### Statistical analyses

Target gene expression was statistically evaluated by comparing ΔCt (Ct value of target gene − Ct value of GAPDH in the same sample) values of different experimental groups. Statistical significance comparing two groups or multiple groups with parametric data was assessed by Student’s *t* test or ANOVA followed by post hoc multiple comparisons (Tukey’s post hoc test). Statistical significance comparing two groups or multiple groups with nonparametric data was assessed by rank sum test or ANOVA on the ranks. A *p* value of < 0.05 was considered statistically significant. Data are presented as scatter plots with mean and standard deviations highlighted.

## Results

### PRG4 reduced *ACTA2* expression and stress fiber formation in osteoarthritic fibroblast-like synoviocytes (OA FLS) and CD44 was involved in the uptake of rhPRG4 by OA FLS, whereas rhPRG4-CD44 interaction affected *ACTA2* expression in response to TGF-β

**Fig. 1:**
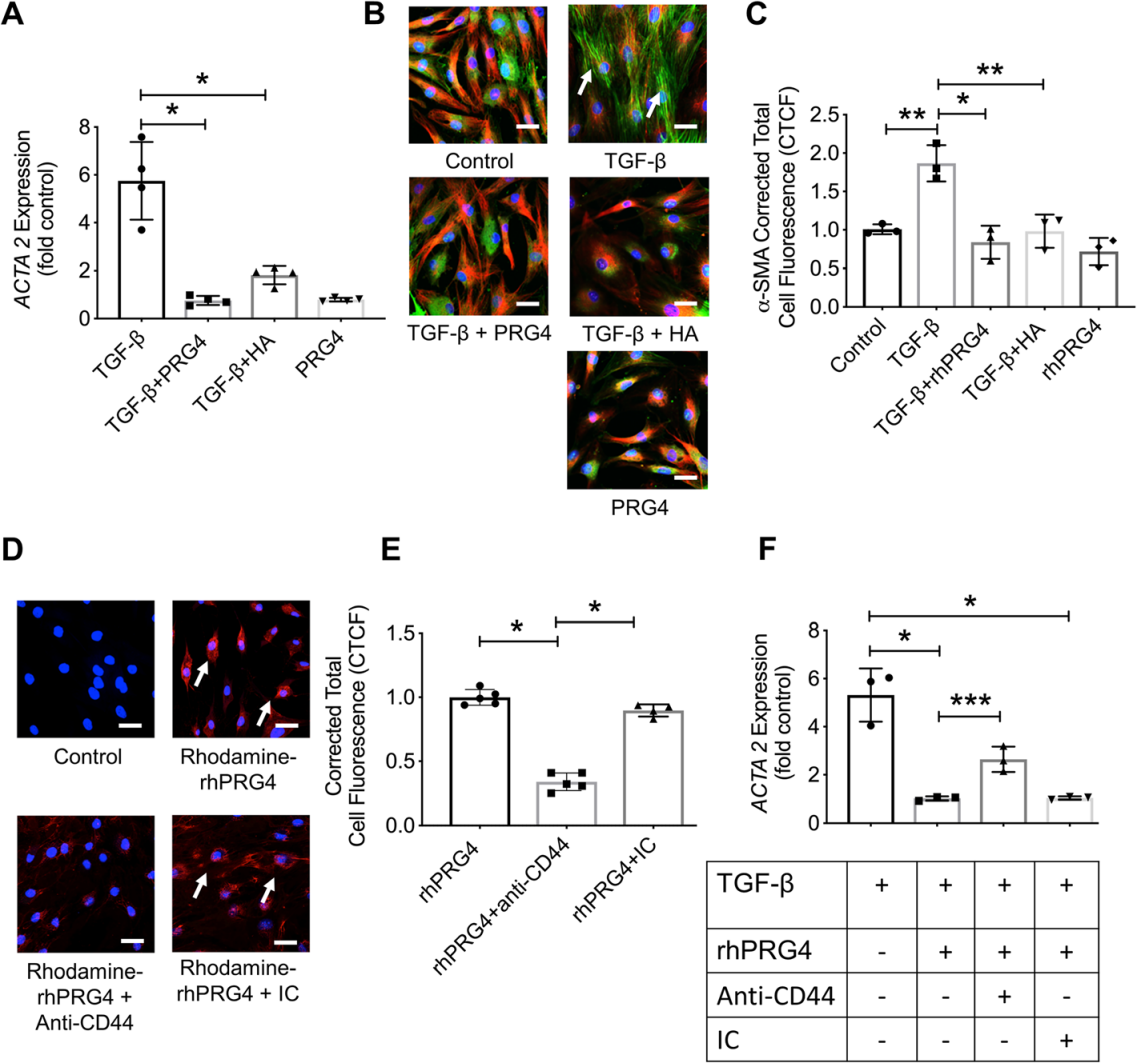
Impact of proteoglycan-4 (PRG4) and high-molecular weight hyaluronic acid (HA) treatments on TGF-β1-induced alpha smooth muscle actin (α-SMA) expression (*ACTA2*), production and stress fiber formation in fibroblast-like synoviocytes isolated from patients with OA (OA FLS) undergoing knee replacement surgery and CD44-dependent uptake of rhodamine-labeled recombinant human PRG4 (rhPRG4) into OA FLS and associated regulation of *ACTA2* expression. OA FLS were treated with TGF-β1 (1 ng/mL) ± rhPRG4 (~ 240 kDa) or HA (~ 1200 kDa) (100 μg/mL for both treatments) for 24 h followed by RNA isolation and qPCR using *GAPDH* as an internal reference gene. OA FLS were stained with anti-α-SMA (green) and counterstained for α-tubulin (red) and DAPI (blue). Corrected total cell fluorescence (CTCF) of α-SMA was determined following a 48-h treatment, and normalized to controls. Rhodamine-labeled rhPRG4 was incubated with OA FLS ± anti-CD44 or isotype control (IC) antibodies for 30 min, and CTFC was quantified. OA FLS were treated with TGF-β1 ± rhPRG4 ± anti-CD44 or IC for 24 h and *ACTA2* expression was determined. Data are presented as a scatterplot with means and standard deviations highlighted utilizing OA FLS from 3 to 4 different patients. *p < 0.*001*; ***p < 0.*05*. Scale = 50 μm. **a** PRG4 and HA reduced *ACTA2* expression in OA FLS. **b** Representative images showing TGF-β1-induced stress fiber formation in OA FLS (white arrows) and PRG4 or HA treatments prevented their formation. **c** PRG4 and HA reduced α-SMA CTCF in OA FLS. **d** Representative images showing rhodamine-rhPRG4 intracellular localization (white arrows), following incubation with OA FLS. **e** Co-incubation of rhPRG4 and anti-CD44 reduced cellular uptake of rhPRG4. **f** Anti-CD44 antibody co-treatment reduced rhPRG4’s antifibrotic effect in OA FLS

PRG4 and HA treatments demonstrated equivalent efficacies in reducing *ACTA2* expression in OA FLS (Fig. 1a). Both treatments reduced *ACTA2* expression compared to the positive control TGF-β1 (*p < 0*.*001* for TGF-β1 + PRG4 or TGF-β1 + HA against TGF-β1 alone). In addition to reducing *ACTA2* expression, PRG4 reduced α-SMA content in OA FLS (*p < 0*.*001* versus TGF-β1 alone; Fig. 1c). A similar effect was also observed for HA treatment (*p < 0*.*001* versus TGF-β1 alone; Fig. 1c). The effect of PRG4 was biologically significant with approximately 52% reduction in mean α-SMA immunofluorescence compared to TGF-β1 alone. In addition to reducing α-SMA in OA FLS, PRG4, and HA treatments prevented the formation of stress fibers in OA FLS in response to TGF-β1 stimulation (Fig. 1b). rhPRG4 was rapidly internalized by OA FLS and the mechanism of its internalization was related to its interaction with CD44. This was illustrated by a reduction in OA FLS CTCF with an anti-CD44 treatment (Fig. 1d, e). Neutralization of CD44 receptor did not completely block rhPRG4 uptake by OA FLS (Fig. 1e). Alternatively, treatment with an IC antibody did not alter rhPRG4’s uptake by OA FLS (*p > 0*.*05*; Fig. 1e). Inhibition of *ACTA2* expression by rhPRG4 was attenuated by pre-incubation with an anti-CD44 antibody (*p < 0*.*05* for TGF-β1 + rhPRG4 + anti-CD44 versus TGF-β1 + rhPRG4; Fig. 1f). In contrast, IC antibody treatment did not alter the magnitude of reduction in *ACTA2* expression observed with rhPRG4 (Fig. 1f).

### CD44 receptor facilitated rhPRG4 uptake by HEK-TGFβ cells and rhPRG4 inhibited TGF-β1/Smad pathway activation

**Fig. 2:**
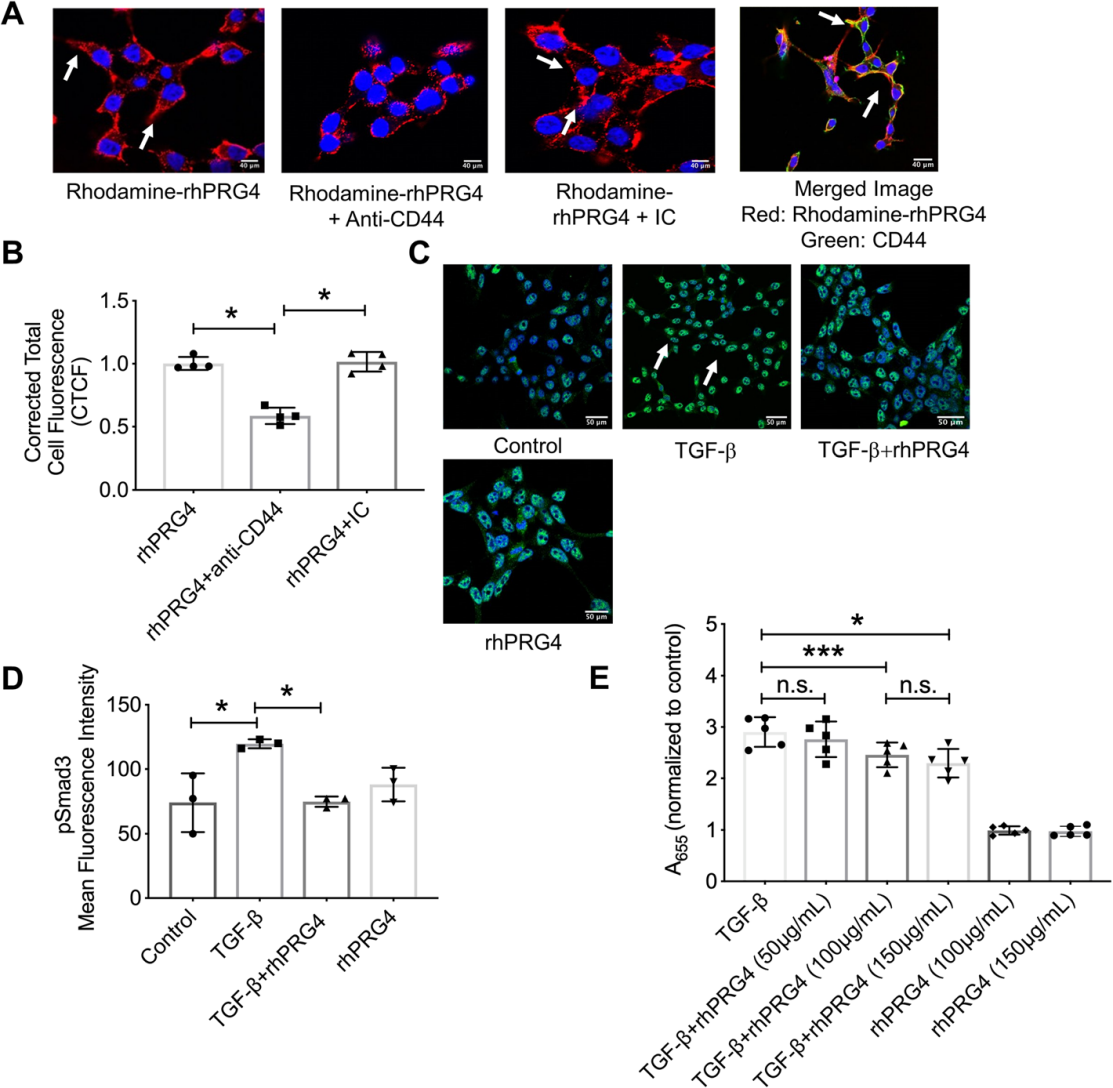
CD44-dependent interaction of recombinant human proteoglycan-4 (rhPRG4) with HEK Blue-TGF-β cells and associated modulation of phosphorylated Smad3 (pSmad3) and TGF-β/Smad signaling pathway. HEK Blue-TGF-β is an engineered cell line produced by transfecting human embryonic kidney (HEK) cells with TGF-β receptor 1 (TGF-β R1), Smad3, and Smad4 genes. Activation of TGF-β R1 results in expression of secreted alkaline phosphatase (SEAP) whose activity can be determined colorimetrically. Rhodamine-labeled rhPRG4 was incubated with HEK Blue-TGF-β cells ± anti-CD44 or isotype control (IC) antibodies and corrected total cell fluorescence (CTCF) was determined. pSmad3 immunocytostaining was performed using an antibody against pSmad3 and pSmad3 fluorescence intensity was determined. Activity of TGF-β/Smad pathway was determined colorimetrically. *p < 0.*001*; **p < 0.*01*; n.s., non-significant. Scale in **a** = 40 μm; scale in **c** = 50 μm. **a** Representative images showing rhPRG4 internalization (white arrows) by HEK-TGF-β cells and co-localization with CD44 receptor (white arrows in merged image). **b** rhPRG4 uptake by HEK-TGF-β cells was reduced by CD44 receptor neutralization. **c** Representative images showing pSmad3 staining in HEK-TGF-β cells following TGF-β stimulation (white arrows) ± rhPRG4 treatment. **d** pSmad3 immunocytostaining was reduced following rhPRG4 treatment. **e** rhPRG4 treatment dose-dependently reduced TGF-β/Smad signaling pathway in TGF-β-stimulated HEK-TGF-β cells

Representative images demonstrate intracellular red fluorescence following the incubation of rhodamine-labeled rhPRG4 with HEK-TGFβ cells (Fig. 2a). Antibody-mediated CD44 receptor neutralization reduced the intensity of red fluorescence, indicative of reduced rhPRG4 uptake into HEK-TGFβ cells (*p < 0*.*001* vs. rhPRG4 alone; Fig. 2a, b). This effect was specific to CD44 receptor, as a non-specific IC antibody pre-incubation did not alter the extent of rhPRG4 uptake into HEK-TGFβ cells (*p > 0*.*05*; Fig. 2a, b). Further evidence of CD44 involvement in rhPRG4 uptake by HEK-TGFβ cells is provided by the intracellular co-localization of rhPRG4 (red) and CD44 (green) as shown by arrows (yellow color) (Fig. 2a). TGF-β1 stimulation resulted in Smad3 phosphorylation as shown in representative images (Fig. 2c). Intense green fluorescence in TGF-β1-stimulated HEK-TGF-β cells indicated the formation of pSmad3. rhPRG4 treatment reduced mean pSmad3 staining intensity subsequent to TGF-β1 stimulation (*p < 0*.*001*; Fig. 2d). In addition, rhPRG4 treatment dose-dependently reduced TGF-β1/Smad pathway activation as shown by a reduction in the formation of SEAP colored product (*p < 0*.*05* for 100 and 150 μg/mL concentrations versus TGF-β1 alone; Fig. 2e).

### TGF-β induced the formation of stress fibers and FAs in murine fibroblast NIH3T3 cells resulting in enhanced cell migration, and these effects were reduced with rhPRG4 treatment

**Fig. 3:**
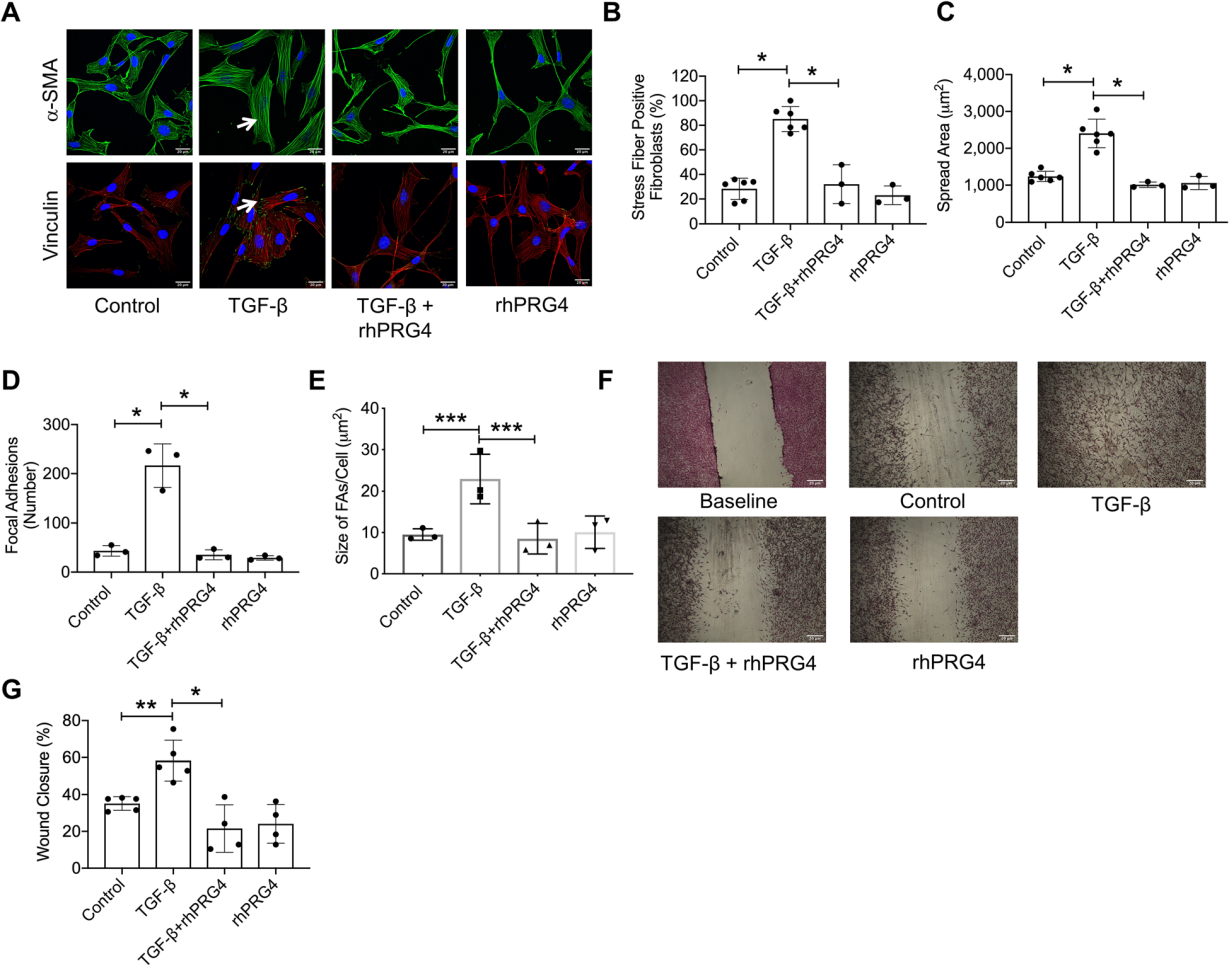
Impact of recombinant human proteoglycan-4 (rhPRG4) treatment on TGF-β induced stress fiber formation, vinculin expression, and formation of focal adhesions (FAs) in murine fibroblasts (NIH3T3) and its relationship to cell migration. Stress fiber formation was probed using an anti-alpha smooth muscle actin (α-SMA) antibody (green) and a blinded investigator evaluated % stress fiber-positive fibroblasts. Vinculin (a marker of FAs) expression was probed using an anti-vinculin antibody (green) and cells were counterstained using rhodamine-labeled phalloidin (cytoskeleton label; red). NIH3T3 Fibroblast migration was performed using a scratch assay and the wound closure percentage was determined. *p < 0.*001*; **p < 0.*01*; ***p < 0.*05*; n.s., non-significant. Scale in **a** and **f** = 20 μm; **a** Representative images showing TGF-β induced stress fiber formation and vinculin staining (white arrows) in NIH3T3 fibroblasts. **b** rhPRG4 reduced stress fiber formation in NIH3T3 fibroblasts. **c** rhPRG4 reduced mean NIH3T3 fibroblast spread area. **d** rhPRG4 reduced the number of FAs in NIH3T3 fibroblasts. **e** rhPRG4 reduced the mean size of FAs in NIH3T3 fibroblasts. **f** Representative images showing NIH3T3 migration in response to TGF-β ± rhPRG4. **g** rhPRG4 reduced NIH3T3 migration

Representative images highlight that murine TGF-β induced the formation of stress fibers and vinculin expression and thus FAs in NIH3T3 cells (Fig. 3a). TGF-β increased the percentage of stress fiber-positive NIH3T3 cells by ~ 5-fold (*p < 0*.*001* against control cells; Fig. 3b) and increased mean cell spread area by ~ 2-fold (*p < 0*.*001* against control cells; Fig. 3c). rhPRG4 treatment reduced the percentage of stress fiber-positive NIH3T3 cells (*p < 0*.*001*; Fig. 3b) and mean cell spread area (*p < 0*.*001*; Fig. 3c) to control levels. TGF-β increased the number of FAs in NIH3T3 cells by ~ 4-fold (*p < 0*.*001*; Fig. 3d), and the mean size of FAs per cell by ~ 2-fold (*p < 0*.*05*; Fig. 3e). Likewise, rhPRG4 treatment reduced the number of FAs (*p < 0*.*001*; Fig. 3d) and mean FA size (*p < 0*.*05*; Fig. 3e) to control levels. A functional outcome of TGF-β induced stress fiber and FAs formation was the enhanced migration of NIH3T3 fibroblasts (Fig. 3f). rhPRG4 reduced TGF-β-stimulated NIH3T3 fibroblast migration (*p < 0*.*001*; Fig. 3g) while rhPRG4 alone did not change basal NIH3T3 fibroblast migration (*p > 0*.*05* against control; Fig. 3g).

### Lack of *Prg4* expression in murine synovial fibroblasts was associated with increased formation of stress fibers, mean FA size, and enhanced basal cell migration, which was reduced by rhPRG4 or anti-CD44 antibody treatments

**Fig. 4:**
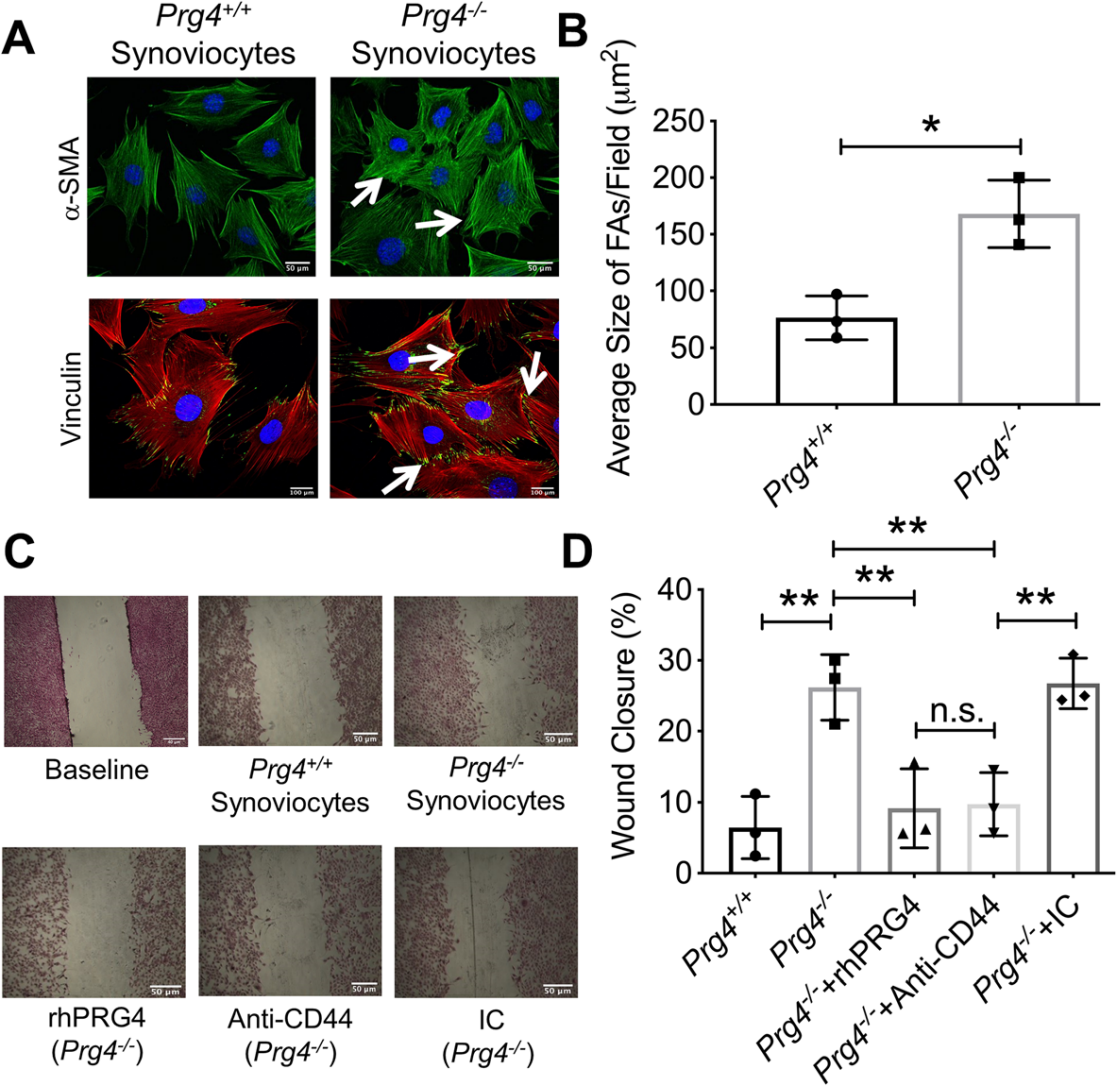
Stress fibers and focal adhesions (FAs) in murine synovial fibroblasts isolated from *Prg4*^*−/−*^ and *Prg4*^*+/+*^ mice and their relationship to cell migration. Stress fiber formation was probed using an anti-alpha smooth muscle actin (α-SMA) antibody (green). Vinculin (a marker of FAs) expression was probed using an anti-vinculin antibody (green) and cells were counterstained using rhodamine-labeled phalloidin (cytoskeleton label; red). The impact of rhPRG4 treatment (200 μg/mL) on basal *Prg4*^*−/−*^ synovial fibroblast migration was assessed using the scratch assay and the wound closure percentage was determined. To highlight the CD44-dependency of the enhanced basal migration of *Prg4*^*−/−*^ synovial fibroblasts, cells were also treated with either an anti-CD44 or isotype control (IC) antibodies (2 μg/mL for both antibodies). *p < 0.*001*; **p < 0.*01*; n.s., non-significant. Scale in **a** = 50 μm (α-SMA) and 100 μm (vinculin); scale in **c** = 50 μm. **a** Representative images showing increased stress fibers and FAs in *Prg4*^*−/−*^ synoviocytes. **b***Prg4*^*−/−*^ synoviocytes demonstrated a higher mean FA size than *Prg4*^*+/+*^ synoviocytes. **c** Representative images showing enhanced basal migration of *Prg4*^*−/−*^ synoviocytes compared to *Prg4*^*+/+*^ synoviocytes. **d** rhPRG4 and anti-CD44 treatments were equally effective in reducing basal migration of *Prg4*^*−/−*^ synoviocytes

Representative images demonstrate enhanced α-SMA and vinculin staining in *Prg4*^*−/−*^ synoviocytes compared to *Prg4*^*+/+*^ synoviocytes (Fig. 4a). The mean size of FAs was ~ 2-folds higher in *Prg4*^*−/−*^ synoviocytes compared to *Prg4*^*+/+*^ synoviocytes (*p < 0*.*001*; Fig. 4b). *Prg4*^*−/−*^ synoviocytes displayed enhanced basal migration compared to *Prg4*^*+/+*^ synoviocytes (*p < 0*.*01*; Fig. 4c, d). rhPRG4 and anti-CD44 antibody treatments reduced *Prg4*^*−/−*^ synoviocyte migration (*p < 0*.*01 for both treatments*; Fig. 4d) with no significant difference between both treatments (*p > 0*.*05*).

### LPS stimulation of macrophages increased active and total TGF-β levels in a macrophage and fibroblast co-culture model, induced fibroblast migration and rhPRG4 treatment reduced fibroblast migration without altering active or total TGF-β levels

**Fig. 5:**
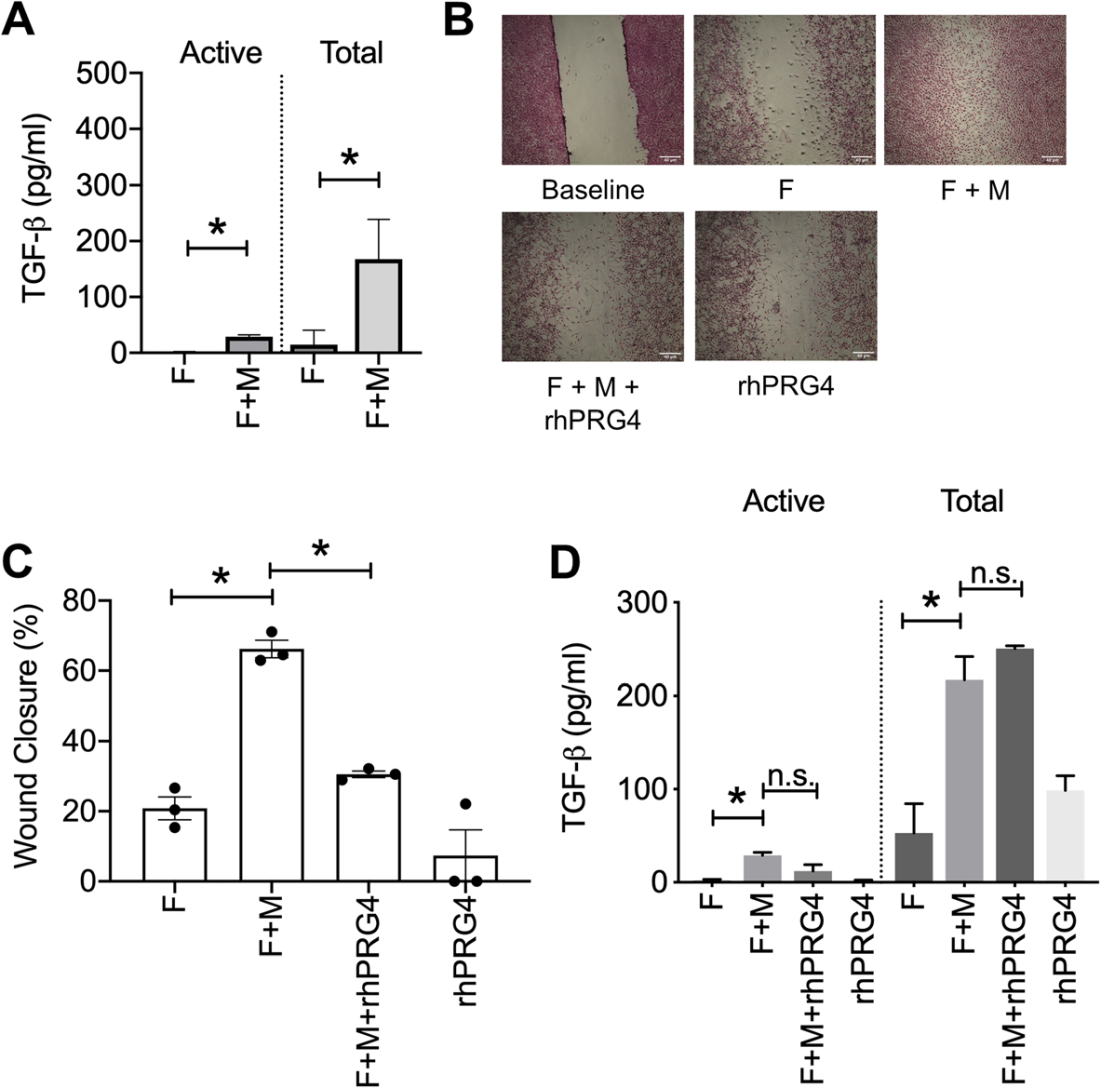
Impact of co-culturing murine fibroblasts (F) and murine macrophages (M) on active and total supernatant TGF-β levels, fibroblast migration, and efficacy of rhPRG4 in regulating fibroblast migration in the co-culture model. Murine macrophages (J774A) were stimulated with lipopolysaccharide (LPS; 5 μg/mL) for 24 h prior to seeding in the top chamber of a co-culture system. Murine fibroblasts (NIH3T3) were cultured in the lower chamber of the same system. Active and total TGF-β media levels were determined using an ELISA. A scratch was performed in the fibroblast monolayer and fibroblast migration in the lower chamber was determined at 48 h ± rhPRG4 (200 μg/mL). *p < 0.*001*; **p < 0.*01*; n.s., non-significant. Scale = 40 μm. **a** Active and total TGF-β concentrations were higher in the fibroblast and macrophage co-culture compared to fibroblasts alone. **b** Representative images of fibroblast migration across different experimental groups. Fibroblast migration was highest in co-cultured fibroblasts and macrophages and rhPRG4 treatment reduced fibroblast migration. **c** rhPRG4 reduced fibroblast migration in a fibroblast and macrophage co-culture model. **d** rhPRG4 did not alter active or total TGF-β media levels in the fibroblast and macrophage co-culture model

In response to LPS stimulation and co-culture of macrophages (M) and fibroblasts (F), active TGF-β levels in the macrophage and fibroblast co-culture were higher compared to fibroblasts alone (*p < 0*.*001*; Fig. 5a). Correspondingly, total TGF-β levels in the macrophage and fibroblast co-culture were higher compared to fibroblasts alone (*p < 0*.*001*; Fig. 5a). The increase in active and total TGF-β levels resulted in enhanced fibroblast migration in the macrophage and fibroblast co-culture compared to fibroblasts alone (*p < 0*.*001*; Fig. 5b, c). rhPRG4 treatment reduced fibroblast migration in the co-culture model (*p < 0*.*001*; Fig. 5c). The effect of rhPRG4 was not mediated by an alteration in active or total TGF-β levels, shown by the lack of differences in active or total TGF-β concentrations between rhPRG4-treated and untreated cells (*p > 0*.*05*; Fig. 5d).

### Lack of *Prg4* expression resulted in progressive synovial tissue fibrosis, shown by enhanced α-SMA, collagen type-I, and PLOD2 production and *Prg4* re-expression reduced synovial tissue fibrosis and this effect was evident in CD44-deficient mice

**Fig. 6:**
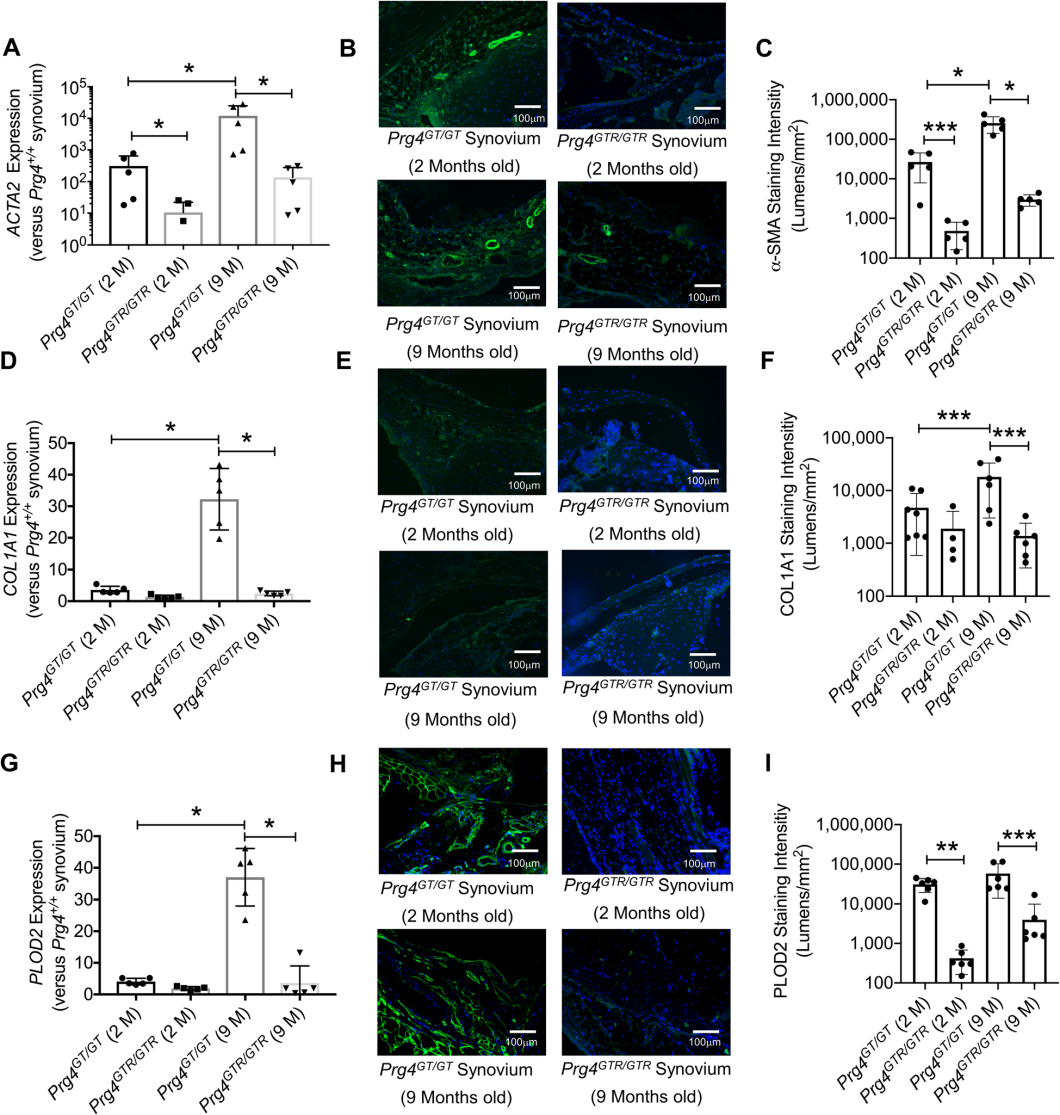
Gene expression and immunohistological analysis of fibrotic markers: alpha smooth muscle actin (α-SMA) (*ACTA2*), collagen type-1 (*COL1A1*) and procollagen-lysine, 2-oxoglutarate 5-dioxygenase (*PLOD2*) in synovial tissues isolated from 2- and 9-month-old gene-trap (*Prg4*^*GT/GT*^) and 2- and 9-month-old recombined gene-trap (*Prg4*^*GTR/GTR*^) mice. The *Prg4* gene-trap allele is a loss of function allele (*Prg4*^*GT/GT*^) whose function is restored with *Cre*-excision (*Prg4*^*GTR/GTR*^). Recombination occurred at 3 weeks of age using intraperitoneal tamoxifen (0.1 mg/g in 100 μL corn oil vehicle) daily for 10 days. Fibrotic markers were probed using specific antibodies and staining intensities (lumens per square millimeter) were quantified. *p < 0.*001*; **p < 0.*01*; ***p < 0.*05*. Scale = 100 μm. **a***ACTA2* expression was higher in 9-month *Prg4*^*GT/GT*^ compared to 2-month littermates and was reduced by *Prg4* re-expression. **b** Representative images showing enhanced α-SMA immunostaining in 2- and 9-month *Prg4*^*GT/GT*^ synovia. **c***Prg4* re-expression reduced α-SMA content in 2- and 9-month-old animals. **d***COL1A1* expression was higher in 9-month-old *Prg4*^*GT/GT*^ animals compared to 2-month and was reduced by *Prg4* re-expression. **e** Representative images showing enhanced collagen type-I immunostaining in 9-month-old *Prg4*^*GT/GT*^ synovia. **f** Collagen type-I content was higher in 9-month-old *Prg4*^*GT/GT*^ animals and was reduced with *Prg4* re-expression. **g***PLOD2* expression was higher in 9-month-old *Prg4*^*GT/GT*^ animals compared to 2-month and was reduced by *Prg4* re-expression. **h** Representative images showing enhanced PLOD2 immunostaining in *Prg4*^*GT/GT*^ synovia. **i***Prg4* re-expression reduced PLOD2 content in *Prg4*^*GT/GT*^ synovia in 2- and 9-month-old animals

Synovial tissues from *Prg4*^*GT/GT*^ animals displayed higher *ACTA2*, *COL1A1*, and *PLOD2* expression levels compared to *Prg4* wildtype synovia (*p < 0*.*001* for all comparisons; Fig. 6a, d, g). Interestingly, lack of *Prg4* expression resulted in a greater increase in *ACTA2* expression compared to *COL1A1* and *PLOD2* (*p < 0*.*001* for all comparisons). The expression of fibrotic markers in synovial tissues from *Prg4*^*GT/GT*^ animals progressed with age. *ACTA2*, *COL1A1*, and *PLOD2* expression levels in 9-month-old *Prg4*^*GT/GT*^ animals were higher than corresponding expression levels in 2-month-old *Prg4*^*GT/GT*^ animals (*p < 0*.*001* for all comparisons). The regulation of fibrotic gene expression in the synovium by PRG4 was further confirmed by the reduction in *ACTA2*, *COL1A1*, and *PLOD2* expression in 9-month-old *Prg4*^*GTR/GTR*^ animals compared to age-matched *Prg4*^*GT/GT*^ animals (*p < 0*.*001* for all comparisons). Representative images of synovial tissues stained for α-SMA, collagen type-I, and PLOD2 proteins are shown in Fig. 6b, e, and h. There was no significant difference in mean collagen type-I staining intensity between 2-month-old *Prg4*^*GT/GT*^ and age-matched *Prg4*^*GTR/GTR*^ animals (*p > 0*.*05*; Fig. 6f). Alternatively, mean α-SMA, collagen type-I, and PLOD2 staining intensities were lower in 9-month-old *Prg4*^*GTR/GTR*^ animals compared to age-matched *Prg4*^*GT/GT*^ animals (*p < 0*.*05 for all comparisons*; Fig. 6c, f, i).

**Fig. 7:**
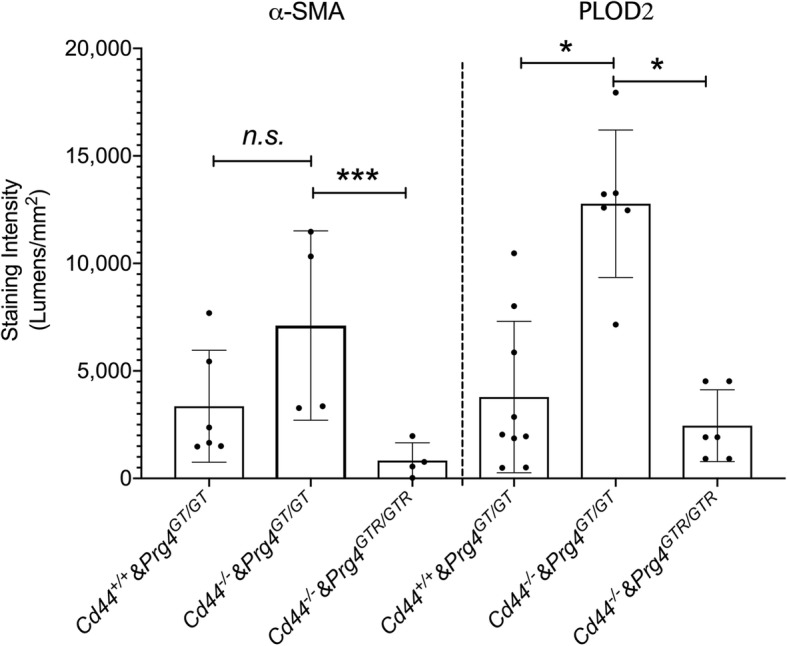
Modulation of expression of fibrotic markers: alpha smooth muscle actin (α-SMA) and procollagen-lysine, 2-oxoglutarate 5-dioxygenase (PLOD2) in synovial tissues from *Prg4* gene-trap animals by CD44. The *Prg4* gene-trap allele is a loss of function allele (*Prg4*^*GT/GT*^) whose function is restored with *Cre*-excision (*Prg4*^*GTR/GTR*^). *Prg4*^*GT/GT*^ animals were crossed with *Cd44*^*−/−*^ mice to generate *Prg*^*GT/GT*^&*Cd44*^*−/−*^ animals. Recombination occurred at 3 weeks of age using intraperitoneal tamoxifen (0.1 mg/g in 100 μL corn oil vehicle) daily for 10 days. Fibrotic markers were probed using specific antibodies and staining intensities (lumens per square millimeter) were quantified. *Prg4* recombination reduced α-SMA and PLOD2 staining in synovial tissues of 2-month-old *Cd44*^*+/+*^ and *Cd44*^*−/−*^ animals. *p < 0.*001*; ***p < 0.*05*; n.s., non-significant

The contribution of CD44 to PRG4’s effect on α-SMA and PLOD2 expression was further studied in vivo by comparing histological staining intensities in synovial tissues from *Cd44*^*+/+*^&*Prg4*^*GT/GT*^, *Cd44*^*−/−*^&*Prg4*^*GT/GT*^, and *Cd44*^*−/−*^*&Prg4*^*GTR/GTR*^ (Fig. 7). While mean α-SMA staining intensity trended higher in *Cd44*^*−/−*^&*Prg4*^*GT/GT*^ synovia compared to *Cd44*^*+/+*^&*Prg4*^*GT/GT*^ synovia, this increase did not reach statistical significance (*p = 0*.*16*). Mean α-SMA staining intensity in *Cd44*^*−/−*^&*Prg4*^*GTR/GTR*^ synovia was lower than the corresponding mean intensity in *Cd44*^*−/−*^&*Prg4*^*GT/GT*^ synovia (*p < 0*.*05*). The absence of CD44 receptor increased PLOD2 staining in synovial tissues as evidenced by a higher mean staining intensity in *Cd44*^*−/−*^&*Prg4*^*GT/GT*^ animals compared to *Cd44*^*+/+*^&*Prg4*^*GT/GT*^ animals (*p < 0*.*001*). In addition, *Prg4* re-expression reduced mean PLOD2 staining in otherwise *Cd44* null animals (*p < 0*.*001*).

## Discussion

PRG4, also called lubricin and superficial zone protein, is a mucinous glycoprotein with a protein core of 1404 amino acids and a central mucin domain that is extensively glycosylated with O-linked β (1-3) Gal-GalNAc oligosaccharides [29, 30, 50, 51]. PRG4 is secreted from synovial fibroblasts and superficial zone chondrocytes and is found adsorbed on articular surfaces and the synovial membrane in addition to its presence in SF [52, 53]. PRG4 has a multifaceted role in the joint and one aspect of its role is to provide boundary lubrication, to prevent friction-induced mitochondrial dysregulation and chondrocyte apoptosis [54–56]. Synthesis of PRG4 by cartilage and synovium was reduced in posttraumatic OA (PTOA) animal models and inflammatory cytokines, e.g., IL-1β reduced PRG4 secretion by synovial fibroblasts while TGF-β produced an opposite effect [57–59]. Furthermore, SF PRG4 levels decreased following acute joint trauma and in patients with advanced OA and rheumatoid arthritis (RA) [60–62]. Therapeutically, native and rhPRG4 demonstrated disease-modifying activities in pre-clinical PTOA mediated by their ability to reduce cartilage degeneration, enhance cartilage repair, and reduce chondrocyte apoptosis [42, 63–65].

In this investigation, we studied rhPRG4’s antifibrotic biological effects on fibroblasts in response to exogenously introduced recombinant TGF-β or TGF-β produced by co-cultured macrophages in response to TLR4 receptor stimulation. We identified that PRG4 and HA provide a similar outcome in TGF-β-stimulated OA FLS in the form of preventing transition of fibroblasts to myofibroblasts with inhibition of α-SMA expression and thus stress fiber formation. PRG4 and HA produced an antifibrotic effect in OA FLS using concentrations that are well below what is normally found in SF [60–62]. As HA is the prototypical CD44 ligand, we used HA as a positive control and our findings support that CD44 modulates fibroblast to myofibroblast transition in OA synoviocytes. PRG4 was rapidly internalized by OA FLS, with a prominent role for CD44 in this process. However, CD44 was not the only mechanism by which OA synoviocytes internalized rhPRG4 as approximately 60% of rhPRG4 entry was blocked subsequent to CD44 receptor neutralization. CD44-mediated rhPRG4’s uptake partially contributed to the latter’s antifibrotic role as rhPRG4’s effect on α-SMA expression was significantly weakened, but not completely abolished, when CD44-mediated uptake was blocked. CD44 also played a role in facilitating the enhanced migration of murine synovial fibroblasts that lack *Prg4* expression as antibody-mediated neutralization of the receptor reduced cell migration. This finding is consistent with our prior observation that *Prg4* null synoviocytes had higher CD44 receptor expression levels compared to *Prg4* competent synoviocytes and is supported by an independent finding that CD44 plays a role in facilitating cell migration [39, 66, 67]. Synovial fibrosis developed in *Prg4* null mice and the expression of SMA, PLOD2, and collagen type-I progressively increased as animals aged. Interestingly, the in vivo antifibrotic effect due to *Prg4* re-expression was not dependent on CD44 as endogenously produced PRG4 reduced SMA and PLOD2 expression in *Cd44* null animals.

CD44 is a single-pass transmembrane glycoprotein that is widely expressed in immune and connective tissues, and different isoforms, generated by alternate splicing, have been characterized [66, 67]. The extracellular domain of CD44 binds different ligands and thus can sense a broad array of microenvironmental signals resulting in ligand-specific effects in inflammation, cell migration, and growth [67–69]. In addition to HA and PRG4, other CD44 ligands include osteopontin, chondroitin, fibronectin, and matrix metalloproteinases (MMPs) [69, 70]. The intracellular domain of CD44 lacks intrinsic kinase activity, but it transduces extracellular ligand-specific signals via a combination of proteolytic fragmentation or interactions with different signaling pathways including members of *Src* and Ras family of GTPases and protein phosphatase-2A [69, 71, 72]. HA is cellularly uptaken by CD44, and this interaction mediates HA’s ability to suppress MMP-13 and aggrecanase-1 expression in OA chondrocytes and synoviocytes [73–75]. In addition to inhibiting synovitis, HA treatment inhibited synovial fibrosis in a murine model of TGF-β and treadmill running [75–77]. The effect of HA was CD44-dependent as its antifibrotic effect was abolished in CD44-deficient mice [77]. Contrary to HA, PRG4 had an in vivo antifibrotic effect that was not dependent on CD44. This might be related to PRG4’s ability to regulate other signaling pathways apart from CD44. PRG4 binds to and inhibits the activation of TLR2 and TLR4 receptors by their ligands and by SF aspirates from patients with OA [78, 79]. In addition, PRG4 was internalized by murine macrophages and co-localized with both CD44 and TLR2 receptors in the cytosol of these cells [80]. As such, it is expected that the enhanced TLR2 and TLR4 receptor signaling in the absence of PRG4 would propagate synovitis in *Prg4* null mice. The innate immune-mediated synovitis would in turn contribute to the development of synovial fibrosis. Upon *Prg4* re-expression in 3-week-old animals, PRG4 would attenuate TLR2- and TLR4-mediated synovitis, thereby potentially reducing TLR-regulated fibrotic tissue remodeling [81].

Myofibroblasts are characterized by α-SMA expression, formation of stress fibers, and FAs and are thought to play an effector role in the pathophysiology of tissue fibrosis [81, 82]. Compared to fibroblasts, myofibroblasts produce more collagen and respond to mitogenic signals resulting in cell proliferation, migration, and secretion of matrix-degrading enzymes [81, 83]. While stress fibers and FAs are unique structures within the cell, they are highly interconnected, where they coordinate cell responses to mechanical signals in their microenvironment [84]. Interestingly, the mean size of FAs, and not their molecular composition or density, can accurately predict cell migration [85]. Normal synovial fibroblasts do not appear to express α-SMA, even with TGF-β stimulation [86]. In our study, OA FLS had a basal level of α-SMA expression and in response to TGF-β, α-SMA expression was enhanced and stress fibers appeared. This was also evident in murine NIH3T3 fibroblasts as the transition from a fibroblast to a myofibroblast phenotype was accompanied by stress fiber and FAs formation, with subcellular and cellular changes in the form of increased number and mean size of FAs and cell spread area and thus enhanced cell migration. rhPRG4 acted biologically to reduce the formation of stress fibers and to reduce the mean number and size of FAs in NIH3T3 fibroblasts and this reduction resulted in arresting fibroblast migration. Thus, it is unlikely that the observed antimigratory effect of rhPRG4 was a physical effect consequent to PRG4’s amphipathic nature and its adsorption on hydrophilic and hydrophobic surfaces [87]. The higher mean size of FAs in synoviocytes from *Prg4* null mice, and its resultant enhanced migration argues that PRG4 plays a role in regulating the expression of the cellular machinery mediating migration in vivo. This may have biological relevance in the realm of synovial fibrosis where PRG4 may function to reduce the migration of fibrocytes to the inflamed joint and therefore the likelihood of adding to the pool of existing myofibroblasts in the synovium [88, 89].

Synovial tissue macrophages are critical regulators of the initiation, maintenance, and resolution phases of synovitis [90]. The complex functions of macrophages are enabled by the presence of different macrophage subpopulations within the joint, which fulfill distinct inflammatory and antiinflammatory roles [90]. In synovial tissue fibrosis, bone marrow-derived monocytes and macrophages are found in increased numbers, which may signal their involvement in the development of synovial fibrosis [91, 92]. In response to an inflammatory signal, macrophages produce TGF-β as a regulatory feedback signal to aid in the resolution of inflammation [93, 94]. TGF-β in turn triggers myofibroblast differentiation and production of matrix proteins [38]. In our study, LPS stimulation resulted in increased secretion of TGF-β by macrophages. Cells secrete a latent form of TGF-β, which in turn is proteolytically converted to active TGF-β by a variety of proteases, including MMPs [95]. The generation of active TGF-β in our co-culture system was associated with fibroblast migration. Interestingly, the introduction of rhPRG4 after macrophage activation did not alter the total TGF-β quantity produced by macrophages. Furthermore, rhPRG4 did not alter the rate of activation of latent TGF-β, indicating that the antimigratory effect of rhPRG4 was at the level of fibroblasts’ response to TGF-β receptor activation. This is supported by our observation that rhPRG4 reduced Smad3 phosphorylation in TGF-β reporter cells and that reduction in pSmad3 intracellular levels attenuated the TGF-β/Smad pathway activation. In TGF-β reporter cells, the attenuation of Smad signaling pathway was downstream to rhPRG4 uptake by these cells in a mechanism that involved CD44 receptor, similar to what was observed in OA FLS. However, the precise mechanism that is activated by CD44 engagement that inhibits TGF-β/Smad signaling pathway remains unclear. Another limitation of our study was that we did not study the antifibrotic effect of rhPRG4 in vivo.

## Conclusion

In summary, we demonstrated that PRG4 is an important regulator of synovial tissue fibrosis using a combination of in vitro and in vivo models. PRG4 prevented the transition of human and murine fibroblasts to a myofibroblast phenotype and in OA FLS; the mechanism of PRG4’s antifibrotic effect was partially linked to CD44-mediated cell uptake. rhPRG4 acted biologically to reduce Smad3 phosphorylation, the activation of TGF-β/Smad signaling pathway, and FA mean size which translated to inhibiting fibroblast migration. The lack of *Prg4* expression in murine synovial tissues resulted in enhanced expression of SMA, collagen type-I, and PLOD2, the enzyme responsible for collagen cross-linking. Synovial fibrosis in *Prg4*^*GT/GT*^ animals progressed with age and re-establishing *Prg4* expression was antifibrotic. The role of PRG4 in regulating synovial fibrosis in vivo extends beyond its interaction with CD44 receptor, as *Prg4* re-expression was antifibrotic in *Cd44* null mice. Therapeutically, overexpressing PRG4 in the articular joint, using a viral mediated gene delivery approach, may prove beneficial in reducing synovial fibrosis in addition to reducing cartilage degeneration [96, 97]. The study of PRG4 as an antifibrotic modulator of the joint’s soft tissues is further warranted especially with the known contribution of synovial fibrosis in advanced OA.

## Data Availability

Not applicable.

## Abbreviations

α-SMA: Alpha smooth muscle actin
*ACTA2*: Gene symbol for alpha smooth muscle actin
ANOVA: Analysis of variance
BCS: Bovine calf serum
BSA: Bovine serum albumin
CD44: Cluster determinant 44
*COL1A1*: Gene symbol for collagen type-I
Ct: Cycle threshold
DMEM: Dulbecco’s Modified Eagle Media
ELISA: Enzyme-linked immunosorbent assay
FBS: Fetal bovine serum
GAPDH: Glyceraldehyde-3-phosphate dehydrogenase
OA: Osteoarthritis
OA FLS: Osteoarthritic fibroblast-like synoviocytes
PBS: Phosphate-buffered saline
PCR: Polymerase chain reaction
PLOD2: Procollagen-lysine, 2-oxoglutarate 5-dioxygenase 2
PRG4: Proteoglycan-4
RA: Rheumatoid arthritis
rhPRG4: Recombinant human PRG4
TLR2: Toll-like receptor 2
TLR4: Toll-like receptor 4
